# Firing rate response of neocortical neurons in the fluctuation-driven regime

**DOI:** 10.1186/1471-2202-16-S1-P59

**Published:** 2015-12-18

**Authors:** Yann Zerlaut, Gilles Ouanounou, Bartosz Telenczuk, Charlotte Deleuze, Thierry Bal, Alain Destexhe

**Affiliations:** 1 Unité de Neurosciences, Information et Complexité, CNRS, 91198 Gif sur Yvette, France

## 

Characterizing the input-output properties of neocortical neurons is of crucial importance to understand the properties emerging at the network level. While deriving those input-output relations for artificial neuronal models has been the object of intense investigations, determining those properties for real neocortical cells remains both experimentally and theoretically challenging [1].

Here, we identify four somatic variables that characterize the dynamical state at the soma in the fluctuation-driven regime and we investigate the firing rate response of pyramidal neocortical neurons in this four dimensional space by means of perforated patch recordings in mice cortex in vitro.

We compare our measurements with different single compartment models (Integrate and Fire, Exponential Integrate and Fire and Hodgkin-Huxley models) and we discuss the limitations imposed by those models in the input-output relationship. We also construct an analytical template for the firing rate response and we show that it is able to capture the behavior of both neuronal models and neocortical neurons.

Because of the difficulty to reproduce the measured characteristics with single compartment models, our results argue in favor of phenomenological models for the cellular computation, where the complex details of the biophysical features are absorbed within a flexible higher-level description.

Moreover, the transfer functions obtained from neurons in slices will enable building mean-field models based on realistic neuronal properties.
